# The Impact of Climate Change on Indigenous Arabica Coffee (*Coffea arabica*): Predicting Future Trends and Identifying Priorities

**DOI:** 10.1371/journal.pone.0047981

**Published:** 2012-11-07

**Authors:** Aaron P. Davis, Tadesse Woldemariam Gole, Susana Baena, Justin Moat

**Affiliations:** 1 The Herbarium, Royal Botanic Gardens, Kew, Richmond, Surrey, United Kingdom; 2 Environment and Coffee Forest Forum, Addis Ababa, Ethiopia; University of Western Ontario, Canada

## Abstract

Precise modelling of the influence of climate change on Arabica coffee is limited; there are no data available for indigenous populations of this species. In this study we model the present and future predicted distribution of indigenous Arabica, and identify priorities in order to facilitate appropriate decision making for conservation, monitoring and future research. Using distribution data we perform bioclimatic modelling and examine future distribution with the HadCM3 climate model for three emission scenarios (A1B, A2A, B2A) over three time intervals (2020, 2050, 2080). The models show a profoundly negative influence on indigenous Arabica. In a locality analysis the most favourable outcome is a c. 65% reduction in the number of pre-existing bioclimatically suitable localities, and at worst an almost 100% reduction, by 2080. In an area analysis the most favourable outcome is a 38% reduction in suitable bioclimatic space, and the least favourable a c. 90% reduction, by 2080. Based on known occurrences and ecological tolerances of Arabica, bioclimatic unsuitability would place populations in peril, leading to severe stress and a high risk of extinction. This study establishes a fundamental baseline for assessing the consequences of climate change on wild populations of Arabica coffee. Specifically, it: (1) identifies and categorizes localities and areas that are predicted to be under threat from climate change now and in the short- to medium-term (2020–2050), representing assessment priorities for *ex situ* conservation; (2) identifies ‘core localities’ that could have the potential to withstand climate change until at least 2080, and therefore serve as long-term *in situ* storehouses for coffee genetic resources; (3) provides the location and characterization of target locations (populations) for on-the-ground monitoring of climate change influence. Arabica coffee is confimed as a climate sensitivite species, supporting data and inference that existing plantations will be neagtively impacted by climate change.

## Introduction

Coffee (*Coffea* L.) is the world's favourite beverage and the second-most traded commodity after oil. In 2009/10 coffee accounted for exports worth an estimated US$ 15.4 billion, when some 93.4 million bags were shipped, with total coffee sector employment estimated at about 26 million people in 52 producing countries [1]. Arabica coffee (*Coffea arabica* L.) and robusta coffee (*C. canephora* Pierre ex A.Froehner) are the two main species used in the production of coffee, although the former is by far the most significant, providing approximately 70% of commercial production [1]. The productivity (green bean yield) of Arabica is tightly linked to climatic variability, and is thus strongly influenced by natural climatic oscillations [2]. The stated optimum mean annual temperature range for Arabica is 18–21°C [3], or up to 24°C [4]. At temperatures above 23°C, development and ripening of fruits are accelerated, often leading to the loss of beverage quality [5], although in some locations higher temperatures (24–25°C) can still produce satisfactory yields of beans, such as in northeast Brazil [6]. Continuous exposure to temperatures as high as 30°C leads to stress, which is manifest as depressed growth and abnormalities, such as the yellowing of leaves and growth of tumours on the stem [7]. In regions with a mean annual temperature below 17–18°C growth is also depressed [8]. Occurrence of frosts, even if sporadic, may strongly limit the economic success of the crop [5]. The relationships between climatic parameters and agricultural production is further complicated because these environmental factors influence the growth and the development of the plants in different ways during the various growth stages of the coffee crop [2].

The Intergovernmental Panel on Climate Change [9] predicts that best estimates for average global temperatures, across all scenarios, will be between 1.8°C to 4°C by the end of the twenty-first century. Global temperatures have increased by an average of 0.74°C (+0.56°C to 0.92°C) in the last 100 years (1906–2005), and this increase appears to have accelerated since the 1970s [9]. On this basis it has been forecast that the sustainability of the coffee industry faces serious challenges in the coming decades [2], [10]–[14]. The evidence from coffee farmers, from numerous coffee growing regions around the world, is that they are already suffering from the influences increased warming [11], [14]. Precise modelling of the influence of climate change for either Arabica or robusta is limited. So far work has been largely restricted to climatic envelope forecasting based on optimum and suboptimum (tolerable) temperature and rainfall averages [14], [15], although in Sao Paulo (Brazil) future climatic scenarios have been applied across the federal agricultural zoning system for cultivated Arabica [10].

Indigenous Arabica plays a key role in coffee production in Ethiopia [4] and has an intrinsic value as the storehouse of wild coffee genetic resources, with an estimated value to the coffee industry of 0.5 to 1.5 billion US$ per year [16]. Ethiopia is the fifth largest global exporter of Arabica and the main producer of coffee in Africa; coffee accounts for c. 33% of Ethiopia's total export earnings [1]. The largest and most diverse populations of indigenous (wild) Arabica occur in the highlands of south-western Ethiopia ([4], [17]–[21], but the native range includes satellite population in south-eastern South Sudan (Boma Plateau) and northern Kenya (Mt Marsabit), at altitudes between 950 and 1950 m, although 1200 m is the most frequent lower altitude limit [22]. By comparison, the indigenous distribution of robusta coffee includes much of tropical Africa, from Guinea to western Tanzania, at altitudes of 50–1500 m [22]. The genetic diversity of wild Arabica populations far exceeds that of cultivated varieties used in crop production and accessions held in germplasm collections [21], [23]–[27]. The wild populations also have high functional diversity in terms of disease [28], and pest and drought tolerance [29]–[31]. As part of a future-proofing resource, and especially for providing genetic potential for mitigating climate change, indigenous populations are perceived as a key resource for the medium- to long-term sustainability of Arabica production [16].

In this study we model the indigenous distribution of Arabica for the present day, and for the future under the influence of climate change until 2080, in order to identify priorities (for *in situ* and, and *ex situ* conservation, monitoring, and future research) and facilitate appropriate decision making. We use bioclimatic modelling based on locality data, in combination with future climate change scenarios across predetermined time intervals until 2080. Bioclimatic models typically compare large-scale species distributions (distribution maps) against present-day modelled climate parameters, in order to generate a predictive statistical model [32]. When a model is deemed successful and robust, the future distribution of a species can be projected over a suitable time frame using climate change scenarios, such as those generated by the Met Office Hadley Centre, or the Commonwealth Scientific and Industrial Research Organisation (CSIRO), to provide a future assessment of distribution under changed environmental conditions [32]. Bioclimatic- (or niche-) based modelling has been widely used in the last ten years to predict the potential impacts of climate change on species distributions all over the world [33]. This type of modelling has been judged as ecologically naive by some [34], [35], as species occurrence is dependent not only on climate, but also on ecological processes such as dispersal, colonization, and complex interactions with other organisms. In this respect, process based modelling, including ecological data, are often favoured on theoretical grounds, by injecting ecological realism into the modelling framework [32]. In reality, species-specific process-based modelling remains scarce at the continental and regional scale [33], [36], owing to the difficulties in acquiring and combining data for analysis. It has been pointed out that the use of both approaches, with their own caveats and advantages, are crucial in order to obtain robust results, and that comparisons among models are needed in the near future to gain accuracy regarding predictions of range shifts under climate change [33].

## Materials and Methods

### Ethics

All necessary permits were obtained for the described field studies in South Sudan. Permits and permission for study on the Boma Plateau were agreed and obtained through the John Garang Institute of Science and Technology in Bor, Jonglie State, South Sudan, following agreements with the South Sudanese government. Permission to visit sites in the Boma Plateau National Park was agreed through the Wildlife Conservation Society (WCS), acting on behalf of the Jonglie State government and relevant landowners.

### Mapping data

Locality data for naturally occurring Arabica were derived from three sources: (1) field survey data for populations in Ethiopia (648 datapoints; data collected 2000–2006, T. Gole, unpubl. data); (2) records of wild populations from literature (18 datapoints; from 1941 [37] and 1964/5 [38]); and (3) herbarium specimens (14 datapoints; collections made 1941–1984). Herbarium specimens were surveyed at the Natural History Museum, London (BM), Royal Botanic Gardens, Kew (K), the Museum of Natural History, Paris (P), and the herbarium of the Wageningen branch of the National Herbarium of the Netherlands (WA; [39]). A total of 751 locality records were databased. Data records lacking co-ordinates were geo-referenced using BioGeomancer Workbench (version 1.2.4; http://bg.berkeley.edu/latest [40], paper maps, and in some cases via interview with regional specialists. All co-ordinates, including those that stated the use of a global positioning system (GPS), were carefully verified and/or error-corrected using Google Earth (Version 5; ©2010 Google). Each specimen record was assigned an estimate of confidence, given as the diameter of a circle in km (normally 0.01 to 0.05 km for GPS readings (made at the time of data collection), and 1 to 100 km for estimated historical localities). The field survey data (719 records) provided 89.7% of the data (accuracy up to 50 m diameter); and other data (specimens and literature) 10.3% of the records (9.5%: accuracy of 1–5 km diameter; 0.83%: the six localities mentioned below). Ambiguous records (i.e. those that could not be geo-referenced to a specific locality), and those outside a 5 arc minutes resolution (c. 5 km diameter), were rejected for the purposes of modelling. In total 713 records were used (six localities rejected: three data records with no estimable geo-reference; three with over 5 km diameter accuracy). The accepted data represents 349 unique localities (i.e. no duplication of localities for identical geo-referenced points; referred to as ‘localities’ in the main part of this contribution), were used. ArcGis 10 [41] was used for all mapping outputs.

### Predictive mapping/ecological niche modelling

**Figure 1:**
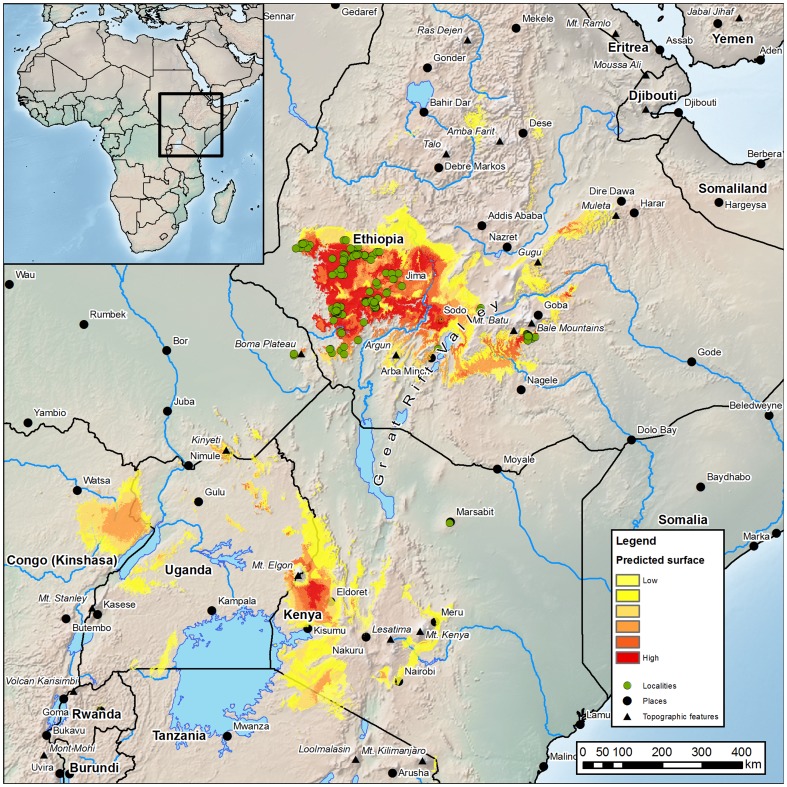
Predicted and actual distribution of indigenous Arabica. Green dots show recorded data-points. Coloured areas (yellow to red) show predicted distribution based on MaxEnt modelling (see internal legend). A context map is given in the top left hand corner.

**Figure 2:**
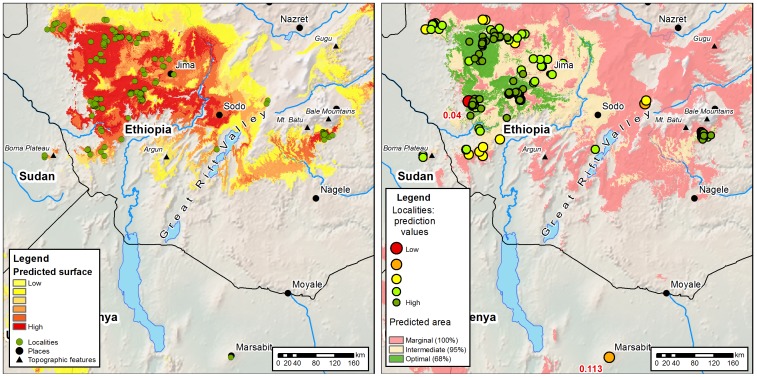
Predicted and actual distribution of indigenous Arabica. Figure 2A. Green dots show recorded data-points. Coloured areas (yellow to red) show predicted distribution based on MaxEnt modelling (see internal legend). Highest predicted area (dark red) indicates ‘core region’. Figure 2B. The same map with thresholds of bioclimatic suitability applied at 68% (optimal), 95% (intermediate) and 100% (marginal) to the localities. Prediction values for each locality are represented by colour and size (see internal legend) with values for low predictions labelled in red, superimposed on predicted surface (space) according to the area analysis. Localities with the smallest (dark green) circles represent ‘core localities’; highest (optimal) predicted area (green) indicates the ‘core region’. See main text for further details.

MaxEnt software (version 3.3.3a) was used to create predictive maps of species occurrence (presence-only; Figures 1 and 2) on the basis of our occurrence data and environmental layers [42]–[44]. MaxEnt has been found to give the best result of all the modelling algorithms available with presence only data [45]–[47] and is frequenly used with herbarium and other collection-based data. This class of environmental (or ecological) niche model (ENM) makes use of a correlative model of the environmental conditions that meet a species ecological requirements and predicts the relative suitability of habitat [48]. The ecological requirements are represented by environmental grids or bioclimatic variables (e.g. temperature, precipitation), which are combined to determine areas and environments that adhere to the species niche, which are manifest as positive areas on the map. The BIOCLIM variables [49] (bioclimatic variables representing annual trends, seasonality and extremes of the environment; accumulated from weather station data from the 1961–1990), accessed via the WorldClim website (http://www.worldclim.org/bioclim. Accessed 2012 Jan 25), were used as grid data at 30 arc seconds resolution (approximately 1 km×1 km). To reduce the processing overhead and data storage, the data was clipped to Africa only. We used the default settings in MaxEnt, which have been shown to give robust and reliable results [44]. The initial models of predicted distribution were tested by omitting 20% of the samples (39 sample points for testing and 158 to produce the model), to gauge the robustness of the models. Area under the curve (AUC), jackknife, analysis of variant contribution, and response curves were also calculated by MaxEnt for further analysis. Preliminary iterations of the models allowed us to identify doubtful outlying localities (i.e. where predictions were very low), and these were corrected where necessary (e.g. geo-referencing errors). A low predictive value was identified for the populations on Mt. Marsabit (Kenya), and so two independent data sets were used, one with and one without Mt. Marsabit.

Spatial sorting bias can give a false impression of model fitness by inflating AUC values [50]. We initially accounted for spatial sorting bias by removing all duplicate localities, (i.e. those with identical georeferences: 713 data records were reduced to 349 unique localities, representing a reduction of 48.9% of the sampling), then these 349 localities were reduced to 197 samples (only one per 1 km grid cell) for the MaxEnt modelling. Then, to more fully test for spatial sorting bias and to check the robustness of the models, further models were produced with spatial sorting bias removed, and for only the core region. Spatial sorting bias was removed by only including points that were separated by at least 0.2 of a degree (c. 22 km), and then checked using ArcGIS 10 [41] average nearest neighbour, which gives a clustering qualification, from clustered, to random, to dispersed. The original 197 samples were highly cluster, but after re-sampling (22 samples points remaining) the points were spatially random. These 22 points were remodelled in MaxEnt, both for the whole of Africa and for a very restricted core region. It should be noted that removal of the bias in this way was only done to test the fitness (comparing AUC and results) of the models; the rest of the analysis was performed using the 349 unique localities (of which 197 were used as the samples for MaxEnt).

Predictive maps represent potential maps of distribution, not the actual distribution of a species: they show where the ecological niche is potentially suitable for the species. The species may not actually occur in this predicted area, for any of the following reasons. (1) The predicted area will include niches that are no longer suitable for the species, because the vegetation has been drastically altered (e.g. deforestation, agriculture). (2) There is niche saturation, e.g. another species or many species already occupy that niche. (3) The models are incorrect due to inaccuracies and weak resolution in the recorded environmental data, which is often the case for microclimates. It should be made clear that the original climate data is also modelled, and based on data from a network of global weather stations. (4) The layers used for modelling are not necessarily the drivers for the occurrence of the species, and other environmental factors (which may drive occurrence) have not been included in the model (e.g. soil characteristics, at a suitable resolution). (5) The locality data is inaccurate. For indigenous Arabica, at least the last two of these points should not strongly influence our distribution model, because the predictive model is very robust (see Results) and the data have been carefully verified by careful scrutiny using satellite maps and ground-truthing, respectively. It should also be said that Arabica coffee is closely tied to narrow environmental parameters, and like the vast majority of coffee species [22] it has a restricted and specific distribution. Data for cultivated Arabica confirms these observations, and shows that this species is sensitive to environmental variables, particularly temperature and precipitation [2], [6], [8]. Soil-water balance is a key factor for the growth of Arabica, although until now this data is only available for the species in cultivation [2], [8]. In addition, observation and reported distribution show that coffee species are rarely adventive, are only scarcely found in secondary (regenerating) vegetation, and quickly become stressed in degraded habitats [22]. In general, the family Rubiaceae includes a very high number of narrowly endemic species, which in most species appears to be related to ecological sensitivity [51].

### Future mapping/climate change modelling

Future predictive maps for indigenous Arabica were generated using the same BIOCLIM layers as employed in the MaxEnt analysis, with predicted future climate data downscaled using the delta method to 30 arc seconds (c. 1 km×1 km). The delta method interpolates the General Circulation Model generally used in climate modelling at scales of 100 to 200 km using a thin plate spline spatial interpolation method to achieve the 30 arc seconds resolution [52]. The data was provided by the CGIAR Research Program on Climate Change, Agriculture and Food Security (CCAFS) (http://www.ccafs-climate.org/statistical downscaling_delta/) [52]. The Met Office Hadley Centre (Hadley Centre for Climate Prediction and Research) climate change model, *Hadley Centre Coupled Model, version 3* (HadCM3) [53], a coupled atmosphere-ocean general circulation model, was used for the time intervals 2020, 2050 and 2080 (note these date represent a time windows of ten years either side of the time interval date, i.e. 2020 is an average of the years 2010–2029, 2050 for 2040–2059 and 2080 for 2070–2089), under three emission scenarios of the IPCC Special Report on Emissions Scenarios (SRES) [54]: scenario A1B (maximum energy requirements; emissions differentiated dependent on fuel sources; balance across sources), A2A (high energy requirements; emissions less than A1/Fl) and B2A (lower energy requirements; emissions greater than B1). We tried, and subsequently rejected, the consensus forecasting with unweighted average approach [55], because it did not provide any further quality to the analyses. There are several climate models available (e.g. HadCM3, CCCMA and CSIRO) but the HadCM3 model was selected for this study because it is presently the only one that spans the years 2020, 2050 and 2080, for all three scenarios, at the resolution of 1 km. It is also reported to provide good median results for Africa compared with other models [13]. Separate model iterations were run with and without Mt Marsabit (Kenya), nine for each iteration, giving a total of 18 models. Locality data (i.e. the 349 unique localities) were queried against all model combinations, in order to provide a value for each of the localities for each of the models.

For the present day models, based on all localities, thresholds were calculated to cut off at 68%, 95% and 100% of the 349 (unique) localities, i.e. based on their relative values within the niche model. As we do not have a totally normal distribution for the prediction at each sample point, 68% is not quite equal to 1 standard deviation. These thresholds were used to classify each Arabica locality though time, within each scenario, into 68% (optimal), 95% (intermediate [suboptimal]; includes the 68% threshold), 100% (marginal [extreme environments]; includes 68% and 95% thresholds). These thresholds were applied to the models to assess the future predicted distribution in terms of bioclimatic suitability (see locality analysis and area analysis, below), with the following assumptions and limitations. (1) Individual plants will not move to different localities that are less suitable than the present day. (2) The area for future modelling was only performed within the core region (i.e. southern Ethiopia and SE South Sudan), in order to reduce the amount of processing and also to limit model spread where there is no control (i.e. no locality data and zero or minimal chance of occurrence). We ran future models including and excluding Mt Marsabit, but either way it made little difference to the modelling. Mt. Marsabit is a considerable distance (c. 500 km) from the main area of Arabica distribution, and is separated from it by a large bioclimatically unsuitable region (low altitude, seasonally dry vegetation). Indigenous Arabica coffee is almost entirely exclusive to the Moist Evergreen Afromontane Forest, and Transitional Rain Forest of Ethiopia [56] and South Sudan [37]. In addition to this, the predictions for occurrence on Mt Marsabit are low. For the future distribution modelling Mt Marsabit was removed from all analyses. (3) It is possible that the Arabica localities used for the modelling are actually now occurring in areas of marginal bioclimatic suitability. This is unlikely given the amount of contemporary locality data we have used, and also because the vast majority of localities have been recorded recently in what is considered to be an optimum environment for the species [56], [57] (S. Demissew, pers. comm.; I. Friis, pers. comm.;T. Gole, pers. observ; .). (4) Vegetation is not included in the modelling; assumptions based on the predictions assume that the vegetation is intact and will remain so until 2080. This does not represent the current situation and future for remaining natural vegetation in the study area. The forest types holding indigenous Arabica are highly fragmented, and zero land-use change from now until 2080 is totally unrealistic. These assumptions were implemented because remaining (actual) vegetation maps for Ethiopia and South Sudan are not available.

### Climate change scenarios (2020–2080) — a locality analysis

**Figure 3:**
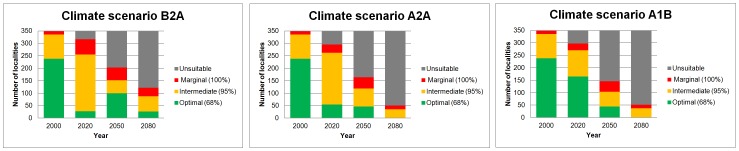
Locality analysis overview I. Predicted climate change outcomes for indigenous Arabica localities for the year interval 2000, 2020, 2050 and 2080. Stacked bar-charts based on Table 1. Green = optimal [bioclimatic] localities (68%); yellow = intermediate (suboptimal) [bioclimatic] localities (95%); red = marginal (extreme) [bioclimatic] localities (100%); grey = unsuitable bioclimatic localities.

**Figure 4:**
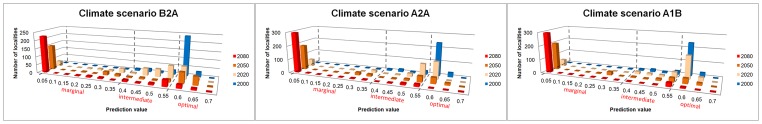
Locality analysis overview II. Predicted climate change outcomes for indigenous Arabica localities (349 in total) for the year intervals 2000, 2020, 2050 and 2080. Histograms for actual predicted values, under each scenario. Dashed line and red text indicate thresholds (68%, 95%, 100%, of the 2000 models). This figure provides finer-scale detail than Figure 3, including the subtle shifts around the thresholds that are evident in the locality analysis. For example in scenario B2A, in 2000 there are a high proportion of localities in optimum bioclimatic space (0.6 and 0.65), but by 2080 most of the localities are outside of all suitable bioclimatic space, with a small number of localities (‘core localites’) still occupying optimal bioclimatic space.

The locality analysis uses actual localities, recorded from 1941 to 2006. As explained above, we used 713 records, representing 349 unique localities (i.e. no duplication of localities for identical geo-referenced points; referred to as ‘localities’ in the main part of this contribution). The localities were used to produce the MaxEnt distribution (reduced to 197 samples for the modelling) for both the locality and area analyses, but in the former the localities were directly analysed against the MaxEnt models through time. For each emission scenario (A1B, B2A, and A2A) a model of future distribution was produced, and then the localities were queried against each scenario and classified into the same thresholds (68%, 95%, 100% and outside 100%) as used for the present day (i.e. the year 2000) predictive models (Figures 3 and 4). For interpretation of the results generated by the locality analysis we need to consider the assumptions used in the downscaling methods when applied to the HadCM3 model [58], which are: (1) changes in climates vary only over large distances, i.e. as large as Global Circulation Model cell size; (2) relationships between variables in the baseline (‘current climates’) are likely to be maintained towards the future. That is, any generalisations or problems in the original BIOCLIM data will be maintained.

To calculate the viability of the localities across all scenarios and dates (including 2000) and as a means of visualizing the ‘core localities’, the totals from the models were calculated for each locality. Where the models return a consistently high predictive value (i.e. where there are high predictions across all scenarios and dates), localities exist in spaces that are predicted to have a relatively constant optimal bioclimatic environment. This provides a simple but powerful means of visualising the data for conservation planning and management. This could be extended by viewing the variability for each locality (to show those localities with highly changeable environments), and providing standard deviations for each locality across all scenarios and dates (data available on request).

### Climate change scenarios (2020–2080) — an area analysis

**Figure 5:**
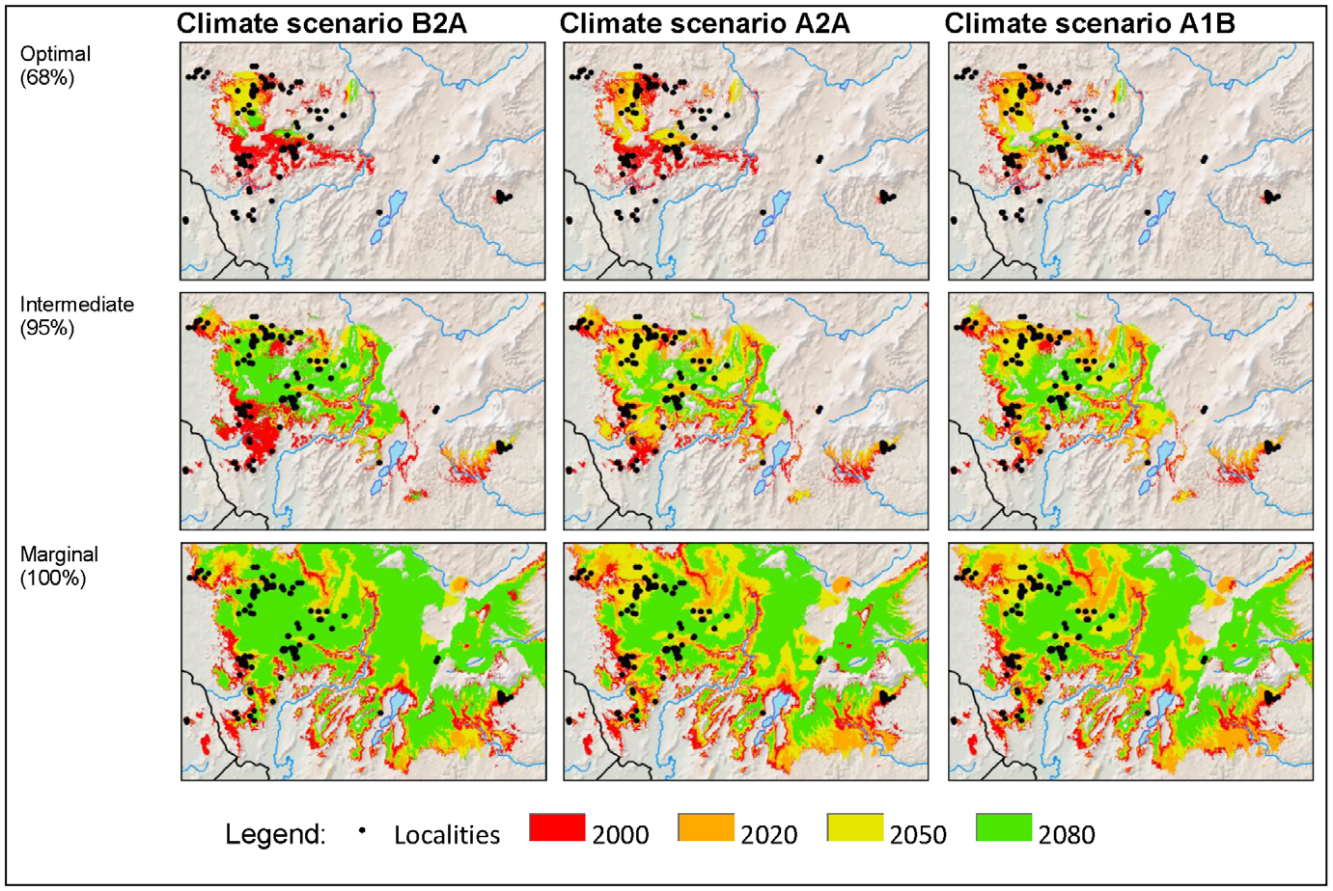
Area analysis maps. Predicted climate change outcomes for indigenous Arabica for the year intervals 2020, 2050 and 2080. Thresholds (of bioclimatic suitability) applied at 68% (optimal), 95% (intermediate) and 100% (marginal). Black dots show historical and present day localities.

**Figure 6:**
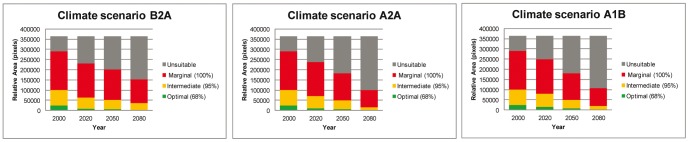
Area analysis overview. Predicted climate change outcomes for indigenous Arabica for the year intervals 2020, 2050 and 2080. Stacked bar-charts based on area analysis maps (Figure 5).

For this analysis each scenario and date map was reclassified using the three thresholds calculated from the original year 2000 threshold percentages (68%, 95% and 100%) to give area-only predicted distribution (suitable bioclimatic space) (Figures 5 and 6). If an area was totally unsuitable in the year 2000, but became suitable in later scenarios, these areas were excluded in the re-classification. Thus, the prediction for 2000 provides a control for future migrations in our modelling (see Discussion – Modelling overview). This assumption was made because significant natural range extension, which requires effective dispersal and colonization, is highly improbable (see Discussion – Implications for wild populations of Arabica coffee).

## Results

### Model of historical and present-day distribution

The potential present-day distribution of indigenous Arabica, as derived from MaxEnt [42]–[44], is shown in Figures 1 and 2, which include the locality records (1941 to 2006), representing the 713 data records, giving the 349 unique localities. For modelling purposes 197 data points were used, as only one data point was retained where points were clustered in a single cell (see Methods). AUC (area under the curve) is often used to evaluate species distribution models [50], [59]. An AUC of 1 represents a perfect prediction and generally values of 0.5 or lower are no better than random, although there are fundamental problems when using AUC for validation [50]. We retrieved an AUC value of 0.99 for the predicted distribution across the whole of Africa, using all 197 sample points; removing spatial sorting bias (i.e. clustering), for the same region gave an AUC of 0.988. Finally, testing of the models after the removal of the spatial sorting bias, and using only the core region (as in Figure 5), gave an AUC value of 0.78. This was performed in order to test the models to the extreme, but not for the actual models used in the rest of the analyses (see Methods). Based on the outcome of the cross-validation tests [50] and expert visual assessment of the predicted distribution we believe that our MaxEnt models were very robust. The distribution includes south-western Ethiopia, south-eastern South Sudan (Boma Plateau) and northern Kenya (Mt. Marsabit). Within Ethiopia, the highest density of recorded presence is in the western-most part of the distribution, and there is a clear division between this area and the eastern side of the Great Rift Valley in the Bale Mountains, although two sites have been recorded between these two main areas of distribution (Figures 1 and 2). Very obvious outlying populations occur on the south-eastern part of Boma Plateau, near Towot (c. 6°6′N 34°26′E), and on Mt. Marsabit (2°16′N 37°58′E). Three localities have very low predictive scores, i.e. Mt. Marsabit (0.11) and two others (0.10 and 0.040; Figure 2B). The latter two localities are physically very close (9.8 and 6.7 km, respectively) to areas with higher predictions. The anomaly could be due to model sensitivity, or data inaccuracies, for example small areas of suitable bioclimatic space that are not picked up by the climate data modelling or the scale of the analyses, such as gallery forest (a forest growing along a watercourse in a region otherwise devoid of trees (or another vegetation type); in many cases gallery forest is less than 1 km wide). Given that these low scoring localities represent a small proportion of the total number of localities, and that the model fit is very strong, they hold little significance in terms of the overall modelling. In addition, even though the Mt. Marsabit locality is only marginally suitable for the occurrence of indigenous Arabica as a whole, there are smaller areas on the mountain that receive a slightly higher prediction than for the entire mountain area (data not shown).

The following BIOCLIM layers give the highest contributions to the model of predicted distribution: bio 4 (Temperature Seasonality); bio 10 (Mean Temperature of Warmest Quarter); bio 14 (Precipitation of Driest Month); and bio 8 (Mean Temperature of Wettest Quarter). Therefore, temperature, precipitation, and their relationship with seasonality, are the biggest drivers for the models, although it should be reiterated (see Methods) that these are not necessarily the actual environmental drivers for the occurrence of the species.

The threshold values (modelled scores given in parentheses) produced for the present day predictive models were 68% of localities (>0.564; ‘optimal’), 95% (>0.392; ‘intermediate’), and 100% (>0.0398; ‘marginal’), as shown in Figure 2B. The optimal localities, i.e. those with 68% or higher, show an excellent visual fit with the actual recorded distribution (the 349 localities), and potential distribution based on specialist knowledge of the vegetation [56], [57] (S. Demissew, pers. comm.; I. Friis, pers. comm.; T. Gole, pers. observ.).

### Climate change scenarios (2020–2080) — a locality analysis

In the locality analysis the future modelled scenarios show a dramatic and profound decrease in the number of predicted bioclimatically suitable localities for indigenous Arabica (Table 1; Figures 3 and 4). In the B2A scenario (lower energy requirements, but with emissions greater than the minimum), which is the most conservative of the scenarios employed, of the 349 localities of 2000 the number is down to 122 by 2080, representing a reduction of 65% of all 349 localities across all thresholds (i.e. 68%, 95% and 100%; Table 1; Figures 3 and 4). At the 68% (optimal) threshold for the B2A scenario, only 26 localities remain suitable, of the original 238 localities from 2000; 212 localities (c. 90%) are outside the 68% threshold, i.e. occurring in either intermediate, marginal, or outside all, suitable bioclimatic localities by 2080. The projections for the localities under other scenarios are even more startling. For A2A (high energy requirements) and A1B (maximum energy requirements with balance across energy sources), by 2080 there are 298 and 299 localities (respectively) outside all thresholds (68%, 95%, 100%) which represents a reduction of 85% of all localities (Figure 3). At the 68% (optimal) threshold for scenarios A2A and A1B the model shows that by 2080 there will be only one locality for each scenario, representing an almost 100% loss of recorded bioclimatically suitable localities.

**Table 1:** Number of (unique) localities in each threshold class for each climate change (emission) scenario and date.

	Present day	Climate scenario B2A			Climate scenario A2A			Climate scenario A1B		
	2000	2020	2050	2080	2020	2050	2080	2020	2050	2080
**Optimal (68%)**	238	27	100	26	54	46	1	165	44	1
**Intermediate (95%)**	97	228	52	62	208	73	34	105	60	36
**Marginal (100%)**	14	61	51	34	33	45	15	27	42	14
**Unsuitable**	0	33	146	227	54	185	299	52	203	298

The locality analysis also shows that in the B2A scenario the number of optimal localities at the 68% threshold dramatically decreases for 2020, but then increases for 2050; and for A2A there is the same dramatic decrease and then a marginal reduction in 2050 (Figure 3). This is partly due to fluctuations around the 65% and 95% threshold values (i.e. around the 0.392 cut off), i.e. moving slightly either way increases/decreases the area of the histogram (see Figure 4 for further detail), and possibly because some localities become more bioclimatically suitable for a short period (e.g. c. 20 years).

### Climate change scenarios (2020–2080) — an area analysis

As in the locality analysis, the area analysis is dominated by a significant reduction in predicted occurrence for Arabica until to 2080. The very inclusive threshold of 100% (marginal, and all other thresholds), where all present day localities would be considered as bioclimatic suitable, even those with very low original prediction values (e.g. 0.0398), still show a 38%, 56% and 55% reduction across all emission scenarios (i.e. B2A, A2A, A1B, respectively). Even under the 95% (intermediate) threshold the A2A and A1B and B2A scenarios show substantial reductions in the distribution area for Arabica, at 57%, 79%, and 75%, respectively (Figures 5 and 6). The prediction under the B2A scenario at the 68% (optimal) threshold is a c. 90% reduction. For A2A and A1B, again with the 68% (optimal) threshold, there is an almost 100% reduction (Figure 6). The area analysis also shows a general northward concentration through time (Figure 5), due to the modelled occurrence of newly available suitable bioclimatic space.

## Discussion

### Modelling overview

Our study shows that modelling driven by locality data of sufficient quantity and quality (i.e. 349 unique localities, each accurate to 30 arc seconds resolution (c. 1 km diameter) or less), and conducted on a regional scale, drives robust models for Arabica. This approach appears to outperform environmental envelope methods based on the climatic thresholds of a limited number of variables [14], [15], [36], e.g. mean temperature and mean rainfall. Our predictive present-day distribution model for Arabica is assumed to be accurate and robust (Figures 1 and 2), due the strength of the distribution model and robust agreement with both ground-truthing and visual assessment using satellite imagery (using Google Earth (Version 5; ©2010 Google). We infer that Mt. Marsabit in northern Kenya is probably not part of the natural range of Arabica, due to the low prediction scores for this locality. This assumption supports the available molecular data, which shows that samples (two in total) from Mt. Marsabit fall within a broad selection of Arabica cultivars and are not aligned with spontaneous populations from Ethiopia [23]. Further work on this outlying locality is required, including fieldwork in those areas that receive slightly higher predictions (see Results – Model of historical and present-day distribution.)

Future distribution predictions for indigenous Arabica based on future scenarios (B2A, A2A, A1B) and the HadCM3 climate model [52], [53] for the time intervals 2020 (an average of the years 2010–2029), 2050 (2040–2059), and 2080 (2070–2089), were analysed using a locality analysis and an area analysis. Both analyses performed well but overall the locality analysis has greater meaning and more practical applications: the data (actual localities based on *in situ* observations across the distribution range) can be tracked through time, from 2020 to 2080. Generally, the locality analysis also requires fewer assumptions. In the area analysis, the 68% (optimal) threshold is likely to be very exclusive. For example, if in 2050 a pixel falls into the 95% (intermediate) threshold from the 68% threshold it would stay within the former threshold in subsequent dates. This same exclusivity is present in the 95% (intermediate) threshold, but to a lesser degree; it does not apply to the 100% (marginal) threshold. The exclusivity of the area analysis means that more caution is required in the interpretation of the predictions. Moreover, in the area analysis the actual area of change is not entirely meaningful, even when surface area values are provided, because of the clipping of the study area and other assumptions made in the modelling. What is important is the relative change across time and scenarios, i.e. the universal reduction of available suitable bioclimatic space until 2080 (Figure 6).

Our modelling approach for Arabica is not constrained by climatic optima, as used in environmental envelope methods [14], [15], [36], and by default encompasses the broader bioclimatic ranges encountered in wild populations, which have a much higher genetic diversity and a greater physiologically variability compared to cultivated Arabica [28], [29]. It is also clear that there is a dichotomy between modelling the success of plantations, which is largely measured by yield and beverage quality [3], [5] and the health and survival of the species, which in stressed environments may exceed the given climatic optima required for successful production of Arabica coffee beans. For example, temperatures above 28–30°C are likely to reduce flower bud formation (and thus fruit production) in indigenous Arabica populations but may not significantly influence morbidity or mortality, at least in the short term (A.Davis and T.Gole, pers. observ.).

Bioclimatic suitability for indigenous Arabica is not a simple association with a linear temperature change but is heavily influenced by seasonality (as identified above). This issue is further complicated by two other factors. Firstly, the present-day prediction (the year 2000) is actually an accumulation of collection dates from 1941–2006 and secondly, the climate data used for the modelling for the year 2000 is accumulated from weather station data from the 1961s to 1990 [49]. The consequence of these considerations is that the prediction for the present-day (2000) distribution of wild Arabica (Figures 1 and 2) could be overly inclusive, that is, the area predicted could be larger than it actually is. However, examination of suitable vegetation and optimal bioclimatic area, based on field observation (S. Demissew, pers. comm.; I. Friis, pers. comm.; T. Gole and A.Davis, pers. observ.) and inspection of satellite imagery shows that this is not the case: the predicted distribution for indigenous Arabica is concurrent with known populations and areas that are highly suitable for the occurrence of this species.

### Implications for wild populations of Arabica coffee

Our modelling shows a profoundly negative trend for the future distribution of indigenous Arabica coffee under the influence of accelerated global climate change. In our locality analysis the most favourable (and most conservative) outcome (scenario B2A; all thresholds) would be a c. 65% reduction in the number of bioclimatically suitable localities, and at worst (scenarios A2A, A1B; 68% threshold) an almost 100% reduction, by the year 2080 (Table 1; Figures 3 and 4). Part of the strength of this analysis is that the locality data used for the modelling covers a high proportion of suitable bioclimatic space in remaining areas of Moist Evergreen Afromontane Forest and Transitional Rain Forest [56], i.e. the vegetation types where Arabica exists. Even if new localities for Arabica are recorded, these are likely to represent a small proportion of those already known, based on the few remaining suitable areas for which we do not have occurrence records. New records are unlikely to influence the modelling, as performed here, to any considerable extent: the predicted percentage loss is unlikely to change dramatically. It should reiterated that our modelling does not incorporate vegetation, due to the absence of a suitable atlas of remaining vegetation for the study area [56]. The assumptions we have made, post modelling, are based on intact vegetation, and on the highly unrealistic premise that there will be negligible human generated land-use change until 2080. Therefore, all of our future predictions should be considered as moderate, at the very least.

In our area analysis, the most favourable outcome (scenario B2A; 100% (marginal) and including all other thresholds) would be a 38% reduction in suitable bioclimatic space, and the least favourable (scenarios A2A, A1B; 68% (optimal) threshold) a 90% reduction, by 2080. The area analysis predicts a general northward concentration through time, i.e. an increase in suitable bioclimatic space in the northern part of the distribution, although the likelihood of migration and establishment by Arabica is assumed to be extremely limited based on insubstantial dispersal and colonization ability, especially in stressed environments. Arabica has a relatively long generation time: even in cultivation it requires a minimum of three to four years to produce fruit and at least five to eight years to reach maximum reproductive potential [60]. Re-colonization potential into suitable areas, let alone marginally suitable ones, is restricted even at the simplest level (e.g. without considering pollinator and dispersal availability, de-forestation, loss of niches to more aggressive colonizers). At best re-colonization will be limited and localized, especially with increasing distance from the parent population. Moreover, it is doubtful that suitable vegetation types will have established within the time-frame required. Totally unsuitable habitat (e.g. *Combretum*-*Terminalia* Woodland and Wooded Grassland) is highly unlikely to become suitable habitat (Moist Evergreen Afromontane Forest, and Transitional Rain Forest [56]) over an 80 year time period. In our methodology we have imposed a zero rate for migration, based on a neutral colonization rate, an assumption that is supported by studies of other forest dwelling plants, where migration rates to newly formed areas of suitable vegetation are given as either nearly impossible [61]–[63] or severely restricted [64]. Birds are probably the main dispersal agents of coffee species in Africa, but modelling indicates that the avian fauna of tropical regions will be reduced in extent and diversity throughout the century [65], and this is likely to reduce the number of possible dispersal events for Arabica. A further compounding factor is that the present coverage of Moist Evergreen Afromontane Forest and Transitional Rain Forest of Ethiopia, the vegetation housing wild populations of Arabica in Ethiopia and South Sudan, is now fragmented, and often degraded [56]. Fragmentation reduces the progress and success of migration for many forest species, and would hinder the establishment of new areas corresponding to Moist Evergreen Afromontane Forest and Transitional Rain Forest vegetation types. Managed relocation of Arabica individuals or even populations by human effort is conceivable, although as with any other form of dispersal a suitable habitat would have to be available during this process and these may be limited (see above) and localized.

In both the locality and area analysis numerous populations outside the main area of distribution (i.e. SW Ethiopia) are predicted to occur outside of all suitable bioclimatic space for Arabica by 2080 (Figures 4 and 5). Even by 2020, some of the populations on the outer edges of the main SW Ethiopia distribution area, several in the Bale Mountains (southern central Ethiopia), and all populations on the Boma Plateau in South Sudan and Mt Marsabit in northern Kenya, will be occurring in unsuitable bioclimatic space. Bioclimatic unsuitability would place populations in high peril, leading to severe stress and a high risk of extinction in the short-term. Ground-truthing would be necessary to test the likelihood and scaling of predictions in terms of population persistence and survival. A recent survey (April 2012) on the Boma Plateau (A.Davis, T. Schilling, S.Krishnan, pers. observ.) is consistent with our modelling. Observations made in the most suitable bioclimatic space on the Boma Plateau indicated that Arabica populations are stressed (loss of aged individuals, meagre population density, minimal seedling recruitment, low-ratio of flower bud development) compared to 70 years ago [37]; subcanopy ambient air temperatures recorded at the end of the dry season (9–12 April 2012), during the middle of the day, were between 28–30°. Some of this stress has no doubt been caused by human intervention, and probably foremost among these would be the burning of the surrounding *Combretum*-*Terminalia* Woodland and Wooded Grassland for grazing, which increases the temperature and lowers the humidity inside the contiguous Transitional Rain Forest. In some places the fire had encroached into the margins of the forest, as demonstrated by the presence of recent charcoal layers detected directly underneath the forest leaf-litter, and this may be expected anywhere where forest and fire-managed grassland co-exist.

There could be a buffer influence for wild populations of Arabica, because the forest micro-environment itself is not included in recorded climate data and therefore not modelled. That is, certain variables such as the mean temperature(s) will be lower inside the forest than in exposed areas in the same general bioclimatic space. In addition, the long generation time of Arabica (c. 50 years, and perhaps up to 100 years [4]) will mean that even if reproduction, dispersal and colonization are reduced, or neutralized, individual trees may persist for some time. However, observations of Arabica populations in South Sudan show that in degraded forest with good canopy cover, at the end of dry season, the difference in ambient air temperature between forest and non forest may only differ by 1°C or less; and on comparing anecdotal accounts with historical records [37] it seems that the mature trees have fared less favourably in these stressed environments compared to juveniles ones (A.Davis, T.Schilling, S.Krishnan, pers. observ.). Moreover, the influences of climate change on the cornerstone species of the Moist Evergreen Afromontane Forest, and Transitional Rain Forest vegetation types are unknown, and thus the outcome for the forest itself cannot yet be predicted.

The impact of multiple compounding influences acting simultaneously on an organism and its associated biota under accelerated climate change would be very difficult to model, but the individual and combined consequence are likely to be negative. The single most important compounding influence for Arabica is almost certainly habitat degradation and loss due to forest modification and clearance, especially for agriculture [4], [17]. This would include the vegetation surrounding and buffering the Moist Evergreen Afromontane Forest and Transitional Rain Forest, i.e. mostly *Combretum*-*Terminalia* Woodland and Wooded Grassland but also Dry Evergreen Afromontane Forest [56]. In particular, *Combretum*-*Terminalia* Woodland and Wooded Grassland is burnt to produce grazing lands [56] and this in turn can raise temperatures and decrease humidity within Moist Evergreen Afromontane Forest and Transitional Rain Forest, and especially in smaller forest fragments.

Pests and diseases are also likely to be important. A study on the East African Kihansi coffee (*C. kihansiensis* A.P. Davis & Mvungi) [66], a species entirely restricted to Kihansi Gorge in Tanzania, provides us with a good example of how coffee species are influenced by pests under accelerated climate change. The underground diversion of the Kihansi River for hydropower production was completed in 1999. The mean temperature and relative humidity at this site in 1997 were 21.23°C and 76.64%, respectively; six years after dam construction these had changed to 24.08°C (+2.84°C) and 68.76% (−7.88%) [67]. These changes coincided with the appearance and chronic spread of a parasitic infestation, apparently correlated to the change in local climate, which has seriously undermined the growth and reproductive potential of this coffee species, with severe consequences for the long-term survival of the species [67]. For Arabica, the coffee berry borer (*Hypothenemus hampei* (Ferrari) [Coleoptera]) poses a significant compounding threat to indigenous populations and plantations. Coffee berry borer, the most important biotic constraint for commercial coffee bean yield worldwide, was unable to complete a single generation per year in SW Ethiopia (Jimma) before 1984, due to low temperatures, but thereafter, because of rising temperatures in the area, it was predicted that the pest would be able to complete one or two generations per year/coffee season [13], [68]. These predictions have been confirmed by an independent study, which shows that the coffee berry borer is now widespread in SW Ethiopia [69]; before 1984 it was absent. Overall, the climatic suitability for coffee berry borer is predicted to increase in southwest Ethiopia [13].

### Implications for cultivated Arabica coffee in Ethiopia and world-wide

The outcome of climate change for Arabica cultivation in Ethiopia, the only coffee grown in the country, is also assumed to be profoundly negative, as natural populations, forest coffee (semi-domesticated) and even plantations occur in the same general bioclimatic space as indigenous Arabica. Forest coffee and semi-forest coffee production systems account for c. 25% of total coffee production in Ethiopia [4]. Production is likely to decrease significantly in certain areas, and especially in locations that are presently marginally suitable for coffee production. Most coffee cultivation in Ethiopia is shade-grown and without irrigation, the latter being a practice that can significantly influence the productivity and survival of Arabica in suboptimal growing areas [60]. Unlike native forests, however, there may be greater short term incentives to employ mitigation measures, such as irrigation, particularly at the lower scales involved (e.g. at farm-level).

Our results provide independent validation that Arabica is a climate sensitive species, supporting data on recorded climate optima [3]–[6], results based on environmental envelope methodologies [14], [15], and anecdotal information from coffee farmers. The logical conclusion is that Arabica coffee production is, and will continue to be, strongly influenced by accelerated climate change, and that in most cases the outcome will be negative for the coffee industry. Optimum cultivation requirements are likely to become increasingly difficult to achieve in many pre-existing coffee growing areas, leading to a reduction in productivity, increased and intensified management (e.g. the use of irrigation), and crop failure (some areas becoming unsuitable for Arabica cultivation). Detailed modelling of Arabica cultivation is required, on local and regional scales, in order to inform famers and decision makers as to the requirements for future-proofing the sustainability of their crop. The methodology used here could be adapted for coffee plantations on a regional scale, by substituting the location of plantations for indigenous populations, and by applying a modified threshold approach based on the parameters encountered and employed in cultivation.

### Conservation of wild Arabica coffee

Unlike cultivated Arabica coffee, the distribution of indigenous populations is controlled almost entirely by natural, biotic parameters, even though these factors are influenced by anthropogenic actions. Assisted migration of wild Arabica could be suggested as a possible means of mitigation, but in reality this option is laden with constraints. Not least are the short-term financial implications associated with resourcing a medium- to long-term and diffuse (i.e. involving multiple populations) action of assisted migration. Re-locating coffee plantations is likely to bring economic benefits within a realistic time frame; the assisted migration of natural populations of Arabica coffee is not.

**Figure 7:**
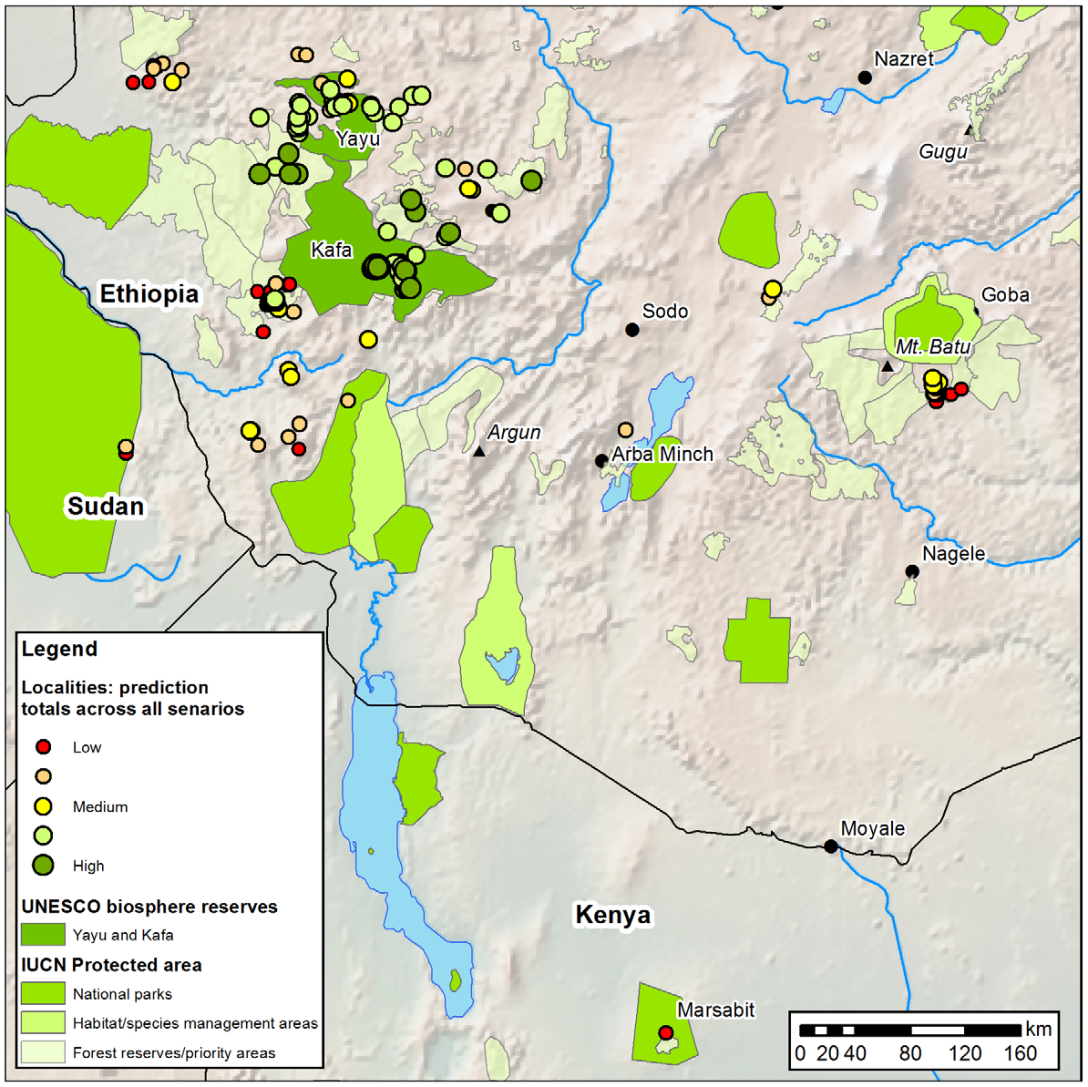
Locality analysis predictions superimposed on main protected areas in the study region. Point size and colour represent the total predicted score for each locality across all scenarios and time intervals (until 2080). Large dots (high score) represent ‘core localities’, i.e. those that predicted to withstand climate change until at least 2080. See internal legend for further details. Protected area data from [70]. Dedicated coffee reserves: Yayu = Yayu Coffee Forest UNESCO MAN Biosphere Reserve (http://www.unesco.org/mabdb/br/brdir/directory/biores.asp?code=ETH02&mode=all. Accessed 2012 May 10), included within the Yayu National Forest Priority Area (NFPA) (http://www.ecff.org.et/component/content/article/10-yayu/6-yayu-coffee-forest-biosphere-reserve.html. Accessed 2012 May 10); Kafa = Kafa Coffee Biosphere Reserve UNESCO Biosphere Reserve (http://www.kafa-biosphere.com/. Accessed 2012 May 10). Note. Controlled Hunting Areas not shown.

What we have shown here is that under a range of emission scenarios some populations of Arabica (occurring in optimal bioclimatic space) might be able to resist climate change until 2080, at least in the absence of severely negative influences (e.g. deforestation). We define these populations here as ‘core localities’ (Figure 7; high prediction totals across all scenarios) and suggest that they should be assessed as candidates for the long-term *in situ* conservation of Arabica in the face of accelerated climate change. Examination of the main protected areas of Ethiopia shows that some of the ‘core localities’ already fall within those established protected areas [70] and have a reasonable to good degree of protection (e.g. national parks and UNESCO biosphere reserves), although many do not (Figure 7). Where there is a specific objective for the *in situ* conservation of indigenous populations of Arabica, such as the Yayu and Kafa Biosphere Reserves (Figure 7), the ‘core localities’ falling closely outside these protected areas should be assessed and, if suitable, be incorporated into protected area delimitation and long-term management. Other ‘core localities’ should be assessed on a case-by-case basis. Conversely, those localities identified as marginally suitable for Arabica in the present-day (Figure 2B) and unsuitable in the short- to medium-term (Figure 7), are suggested as priorities for *ex situ* conservation.

Closely associated with the need to identify populations for conservation will be the requirement to assess the genetic variation of indigenous Arabica, and particularly in relation to their bioclimatic profiles (either modelled or directly measured), and physiological response to climate change. For example, genetic assessment and monitoring of populations either side of the Great Rift Valley could be rewarding, as they are already known to possess different bioclimatic tolerances and other potentially valuable characteristics [30], [31]. These two main areas of distribution receive the bulk of their rainfall from different directions and at different times of the year [57]. In undertaking such work it might be possible to identify local variants that have improved thermal and/or drought tolerance, which can be used in the development of cultivated Arabica stock.

### Next steps for present and future distribution modelling of indigenous Arabica coffee

Despite the limitations of assessing the impacts of climate change using the single-species approach via bioclimatic modelling [32], [33], and in the absence of more detailed ecological and physiological data [34], [35], this study has firmly established a baseline for assessing the consequences of climate change for indigenous Arabica coffee. It is important to bear in mind that we have used here and single climate model (HadCM3), with one niche modelling method (MaxEnt). Whilst the quantitative results could be quite different using other climate and niche models, we believe that the overall projections and trends will be similar with the present resources at hand. For more precise modelling of future distribution trends for Arabica, mapping and modelling work should incorporate actual (remaining) vegetation, as opposed to a potential vegetation [56], although these data are not presently available. These mapping data would be used to ‘clip’ suitable remaining habitat for Arabica from the models, and provide more accurate estimates for loss of suitable bioclimatic space. More detailed ecological data (subcanopy temperatures, soil/water balance, soil type, humidity, evapotranspiration) is also required. Collecting these data across the entire natural range of Arabica is possible, although a focused approach using selected sites is more realistic. On-the-ground monitoring of stress (e.g. seedling recruitment, flower bud and fruit development, leaf yellowing, leaf drop, and pests and disease) for selected populations (as identified here) over suitable time intervals, will be necessary to fully ground-truth the validity and scaling (e.g. categorization of bioclimatic scoring in terms of morbidity and mortality) of our modelling. There is also a requirement to conduct surveys in areas that have high and low predictions for the presence of Arabica (and suitable remaining vegetation) but presently lacking records of occurrence; ideally presence and absence of populations would be accurately recorded in a systematic manner. Finally, as new climate change models are developed, and existing ones refined, there will be a need to re-analyze the existing data, with greater temporal (e.g. 10 year intervals) and spatial resolution, possibly using some sort of consensus approach.

## References

[1] International Coffee Organization (ICO) (2012) Trade Statistics. Available: http://www.ico.org/trade_statistics.asp?section=Statistics. Accessed 2012 Aug 17.

[2] Camargo MBP (2010) The impact of climatic variability and climate change on Arabic coffee crop in Brazil. Bragantia 69: 239–247.

[3] Alègre C (1959) Climates et caféiers d'Arabie. Agron Trop 14: 23–58.

[4] Teketay D (1999) History, botany and ecological requirements of Coffee. Walia, J Ethiopian Wildlife Nat Hist Soc 20: 28–50.

[5] Camargo AP (1985) Florescimento e frutificação de café arábica nas diferentes regiões cafeeiras do Brasil. Pesqui Agropecu Bras 20: 831–839.

[6] DaMatta FM (2004) Exploring drought tolerance in coffee: a physiological approach with some insights for plant breeding. Braz J Plant Physiol 16: 1–6.

[7] Franco CM (1958) Influence of temperature on growth of coffee plant. New York: IBEC Research Institute. 24 p.

[8] DaMatta FM, Ramalho JDC (2006) Impacts of drought and temperature stress on coffee physiology and production: a review. Braz J Plant Physiol 18: 55–81.

[9] IPCC (2007) Summary for policymakers. In Climate Change 2007, published for the Intergovernmental Panel on Climate Change. Cambridge: Cambridge University Press. 18 p.

[10] Zullo J Jr, Pinto HS, Assad ED (2006) Impact assessment study of climate change on agricultural zoning. Meteorol Appl (supplement) 69–80.

[11] Baker PS, Haggar J (2007) Global Warming: the impact on global coffee. SCAA conference handout. Long Beach, USA 14.

[12] Titus A, Pereira GN (2008) Global warming in coffee plantations. Indian Coffee 72: 19–24.

[13] Jaramillo J, Muchugu E, Vega FE, Davis AP, Borgemeister C, et al (2011) Some like it hot: the influence and implications of climate change on coffee berry borer (*Hypothenemus hampei*) and coffee production in East Africa. PLoS ONE 6: e24528. 21935419 10.1371/journal.pone.0024528 PMC3173381

[14] Haggar J, Schepp K (2011) Coffee and climate change. Desk study: impacts of climate change in four pilot countries of the coffee and climate initiative. Hamburg: Coffee and Climate. 78 p.

[15] Ridley FV (2011) The past and future climatic suitability of arabica coffee (*Coffea arabica* L.) in East Africa. Masters thesis, Durham University. Available: http://etheses.dur.ac.uk/680/. Accessed 2012 Jan 3.

[16] Hein L, Gatzweiler F (2006) The economic value of coffee (*Coffea arabica*) genetic resources. Ecol Econ 60: 176–185.

[17] Gole TW (2003) Vegetation of the Yayu Forest in SW Ethiopia: impacts of human use and implications for in situ conservation of wild *Coffea arabica* L. populations. Ph.D. Thesis. University of Bonn, Germany.

[18] Senbeta F (2006) Biodiversity and ecology of Afromontane rainforests with wild *Coffea arabica* L. populations in Ethiopia. Ph. D. University of Bonn, Germany.

[19] Senbeta F, Denich M, Boehmer HJ, Woldemariam T, Teketay D, et al (2007) Wild *Coffea arabica* L. in the Afromontane rainforests of Ethiopia: distribution, ecology and conservation. Ethiopian J Sci 30: 13–24.

[20] Schmitt CB (2006) Montane rainforest with wild *Coffea arabica* in the Bonga Region (SW Ethiopia): plant diversity, wild coffee management and implications for conservation. Ecology and Development Series No. 48. Cuvillier Verlag: Göttingen, Germany.

[21] Labouisse J-P, Bellachew B, Kotecha S, Bertrand B (2008) Current status of coffee (*Coffea arabica* L.) genetic resources in Ethiopia: implications for conservation. Genet Resour Crop Ev 55: 1079–1093.

[22] Davis AP, Govaerts R, Bridson DM, Stoffelen P (2006) An annotated taxonomic conspectus of the genus *Coffea* (Rubiaceae). Bot J Linn Soc 152: 465–512.

[23] Lashermes P, Trouslot P, Anthony F, Combes MC, Charrier A (1996) Genetic diversity for RAPD markers between cultivated and wild accessions of *Coffea arabica* . Euphytica 87: 59–64.

[24] Anthony FM, Bertrand B, Quiros O, Lashermes P, Berthaud J, et al (2001) Genetic diversity of wild coffee (*Coffea arabica* L.) using molecular markers. Euphytica 118: 53–65.

[25] Anthony FM, Combes C, Astorga C, Bertrand B, Graziosi G, et al (2002) The origin of cultivated *Coffea arabica* L. varieties revealed by AFLP and SSR markers. Theor Appl Genet 104: 894–900. 12582651 10.1007/s00122-001-0798-8

[26] Tesfaye T (2006) Coffee forest conservation: local-level institutions influencing the conservation and use of coffee forests in southwest Ethiopia. Kommunikation und Beratung 72. Weikersheim: Margaf Publishers. 187 p.

[27] Dessalegn Y, Herselman L, Labuschagne MT (2008) AFLP analysis among Ethiopian arabica coffee genotypes. Afr J Biotechnol 7: 3193–3199.

[28] Adugna G, Hindorf H, Steiner U, Nirenberg H, Dehne I, et al (2005) Genetic diversity in the coffee wilt pathogen (*Gibberella xylarioides*) populations: differentiation by host specialization and RAPD analysis. J Plant Dis Protect 112: 134–145.

[29] Taye K (2006) Ecophysiological diversity of wild Arabica populations in Ethiopia: growth, water relations and hydraulic characteristics along a climatic gradient. Ecology and Development Series No. 46. Cuvillier Verlag: Göttingen. 305 p.

[30] Burkhardt J, Kufa T, Beining A, Goldbach HE, Fetene M (2007) Drought adaptation strategies of *Coffea arabica* populations along a rainfall gradient in Ethiopia. 21st International Conference on Coffee Science, Montpellier, France, 11–15 September: 1032–1036.

[31] Montagnon C, Bouharmont P (1996) Multivariate analysis of phenotypic diversity of *Coffea arabica* . Genet Resour Crop Ev 43: 221–227.

[32] Ellis CJ (2011) Predicting the biodiversity response to climate change: challenges and advances. Syst Biodivers 9: 307–317.

[33] Morin X, Thuiller W (2009) Comparing niche- and processbased models to reduce prediction uncertainty in species range shifts under climate change. Ecol Lett 90: 1301–1313. 10.1890/08-0134.1 19537550

[34] Hampe A (2004) Bioclimate envelope models: what they detect and what they hide. Global Ecol Biogeogr 13: 469–476.

[35] Sinclair SJ, White MD, Newell GR (2010) How useful are species distribution models for managing biodiversity under future climates? Ecol Soc 15: 8.

[36] Pearson RG, Dawson TP (2003) Predicting the impacts of climate change on the distribution of species: are bioclimate envelope models useful? Global Ecol Biogeogr 12: 361–371.

[37] Thomas AS (1942) The wild Arabica coffee on the Boma Plateau. Anglo-Egyptian Sudan. Empire Journal Expt Agric 10: 207–212.

[38] Meyer FG, Fernie LM, Narasimhaswamy RL, Monaco LC, Greathead DJ (1968) FAO coffee mission to Ethiopia, 1964–1965. Rome: Food and Agriculture Organization of the United Nations. 200 p.

[39] Holmgren PK, Holmgren NH, Barnett LC (1990) Index herbariorum. Part 1: the herbaria of the world, 8th edn. Regnum Vegetabile. New York: New York Botanical Garden. 693 p.

[40] Guralnick RP, Wieczorek J, Beaman R, Hijmans RJ (2006) the BioGeomancer Working Group (2006) BioGeomancer: automated georeferencing to map the world's biodiversity data. PLoS Biol 4: e381. 17105347 10.1371/journal.pbio.0040381 PMC1637066

[41] ESRI (2012) ArcGIS Desktop Release 10. Redlands, CA: Environmental Systems Research Institute.

[42] Phillips SJ, Dudik M, Schapire RE (2004) A maximum entropy approach to species distribution modelling. ACM International Conference Proceeding Series; Vol 69 Proceedings of the 21st International Conference on Machine Learning. New York: ACM Press. pp. 655–662.

[43] Phillips SJ, Anderson RP, Schapire RE (2006) Maximum entropy modelling of species geographic distributions. Ecol Model 190: 231–259.

[44] Phillips SJ, Dudik M (2008) Modeling of species distributions with Maxent: new extensions and a comprehensive evaluation. Ecography 31: 161–175.

[45] Elith J, Leathwick JR (2009) Species distribution models: ecological explanation and prediction across space and time. Annu Rev Ecol Evol Syst 40: 677–697.

[46] Williams JN, Seo CW, Thorne J, Nelson JK, Erwin S, et al (2009) Using species distribution models to predict new occurrences for rare plants. Divers Distrib 15: 565–576.

[47] Mateo RG, Croat TB, Felicísimo ÁM, Muñoz J (2010) Profile or group discriminative techniques? Generating reliable species distribution models using pseudo-absences and target-group absences from natural history collections. Divers Distrib 16: 84–94.

[48] Warren DL, Seifert SN (2011) Ecological niche modeling in MAXENT: the importance of model complexity and the performance of model selection criteria. Ecol Appl 21: 335–342. 21563566 10.1890/10-1171.1

[49] Hijmans RJ, Cameron SE, Parra JL, Jones PG, Jarvis A (2005) Very high resolution interpolated climate surfaces for global land areas. Int J Climatol 25: 1965–1978.

[50] Hijmans RJ (2012) Cross-validation of species distribution models: removing spatial sorting bias and calibration with a null model. Ecology 93: 679–688. 22624221 10.1890/11-0826.1

[51] Davis AP, Govaerts R, Bridson DM, Ruhsam M, Moat J, et al (2009) A global assessment of distribution, diversity, endemism, and taxonomic effort in the Rubiaceae. Ann Mo Bot Gard 96: 68–78.

[52] Ramirez J, Jarvis A (2008) High resolution statistically downscaled future climate surfaces. International Center for Tropical Agriculture (CIAT); CGIAR Research Program on Climate Change, Agriculture and Food Security (CCAFS) Cali, Colombia. Available: http://wwwccafs-climateorg/statistical_downscaling_delta/. Accessed 2012 Jan 3.

[53] Gordon C, Cooper C, Senior CA, Banks H, Gregory JM, et al (2000) The simulation of SST, sea ice extents and ocean heat transports in a version of the Hadley Centre coupled model without flux adjustments. Clim Dynam 16: 147–168.

[54] IPCC (2000) Special Report on Emissions Scenarios (SRES). Working Group III Intergovernmental Panel on Climate Change 21.

[55] Araujo MB, New M (2007) Ensemble forecasting of species distributions. Trends Ecol Evo 22: 42–47. 10.1016/j.tree.2006.09.010 17011070

[56] Friis I, Demissew S, Breugel PV (2010) Atlas of the potential vegetation of Ethiopia. Biol Skrif 58: 1–307.

[57] Friis I (1979) The wild populations of *Coffea arabica* L., and cultivated coffee. In: Kunkel G, editor. Proceedings of the 9th Plenary Meeting of AETFAT: Taxonomic Aspects of African Economic Botany, Las Palmas de Gran Canaria, 18–23 March 1978. Las Palmas de Gran Canaria. pp. 63–68.

[58] Ramirez J, Jarvis A (2010) Downscaling global circulation model outputs: The delta method decision and policy analysis working paper No. 1. International Center for Tropical Agriculture (CIAT); CGIAR Research Program on Climate Change, Agriculture and Food Security (CCAFS). Cali, Colombia. Available: http://www.ccafs-climate.org/media/ccafs_climate/docs/Downscaling-WP-01.pdf.

[59] Franklin J (2010) Mapping species distributions. Cambridge: Cambridge University Press. 338 p.

[60] Wrigley G (1980) Coffee – tropical agriculture series. Harlow: Longman Scientific & Technical. 369 p.

[61] Honnay O, Verheyen K, Butaye J, Jacquemyn H, Bossuyt B, et al (2002) Possible effects of habitat fragmentation and climate change on the range of forest plant species. Ecol Lett 5: 525–530.

[62] Baeten L, Jacquemyn H, Van Calster H, Van Beek E, Devlaeminck R, et al (2009) Low recruitment across life stages partly accounts for the slow colonization of forest herbs. J Ecol 97: 109–117.

[63] Baeten L, De Freene P, Verheyen K, Graae BJ, Hermy M (2010) Forest herbs in the face of global change: a single-species-multiple-threats approach for *Anemone nemorosa* . Plant Ecol Ev 143: 19–30.

[64] Walck JL, Hidayati SN, Dixon KW, Thompson K, Poschlod P (2011) Climate change and plant regeneration from seed. Glob Change Biol 17: 2145–2161.

[65] Jetz W, Wilcove DS, Dobson AP (2007) Projected impacts of climate and land-use change on the global diversity of birds. PLoS Biol 5: e157. 17550306 10.1371/journal.pbio.0050157 PMC1885834

[66] Davis AP, Mvungi E (2004) Two new and endangered species of *Coffea* (Rubiaceae) from the Eastern Arc Mountains (Tanzania) and notes on associated conservation issues. Bot J Linn Soc 146: 237–245.

[67] Rija A, Mwamende KA, Hassan SN (2011) The aftermath of environmental disturbance on the critically endangered *Coffea kihansiensis* in the Southern Udzungwa Mountains, Tanzania. Trop Conserv Sci 4: 359–372.

[68] Jaramillo J, Chabi-Olaye A, Kamonjo C, Jaramillo A, Vega FE, et al (2009) Thermal tolerance of the coffee berry borer *Hypothenemus hampei*: predictions of climate change impact on a tropical insect pest. PLoS ONE 4: e6487. 19649255 10.1371/journal.pone.0006487 PMC2715104

[69] Mendesil E, Jembere B, Seyoum E (2003) Occurrence of coffee berry borer *Hypothenemus hampei* (Ferrari) (Coleoptera: Scolytidae) on *Coffea arabica* L. in Ethiopia. Ethiopian J Biol Sci 2: 61–72.

[70] IUCN, UNEP (2010) The World Database on Protected Areas (WDPA). UNEP-WCMC. Cambridge, UK. Available: www.protectedplanet.net. Accessed 2012 Jan 3.

